# Coronary stenosis is a risk marker for impaired cardiac function on cardiopulmonary exercise test

**DOI:** 10.1186/s12872-022-02935-9

**Published:** 2022-11-14

**Authors:** Siyuan Li, Yifang Yuan, Lanting Zhao, Tingting Lv, Fei She, Fang Liu, Yajun Xue, Boda Zhou, Ying Xie, Yu Geng, Ping Zhang

**Affiliations:** 1 Department of Cardiology, Beijing Tsinghua Changgung Hospital, 168# Litang Road, 102218 Beijing, China; 2 Peking University Clinical Research Center, Peking University First Hospital, Beijing, China; 3 Department of Epidemiology and Biostatistics, Peking University School of Public Health, Beijing, China

**Keywords:** Cardiac function, Cardiopulmonary exercise test, Ischemic heart disease, Atherosclerosis

## Abstract

**Background:**

Cardiac function varies in different ways in ischemic heart disease (IHD). We aimed to evaluate the characteristics of cardiac function on cardiopulmonary exercise test (CPET) in IHD with different coronary stenoses.

**Methods:**

Totally 614 patients with IHD were divided into non-obstructive coronary artery disease (NOCAD) (stenosis < 50%), obstructive coronary artery disease (OCAD) (stenosis 50-90%) and severe OCAD ( stenosis > 90%) according to the coronary angiography. And 101 healthy volunteers as controls. All participants performed CPET to assess cardiac function by oxygen uptake (VO_2_), estimated cardiac output (CO), and heart rate (HR).

**Results:**

Generally, the values of VO_2_, CO, and HR in IHD were significantly lower than in healthy volunteers. Among 289 NOCAD, 132 OCAD, and 193 severe OCAD, significantly decreased values of VO_2_, CO, HR were observed (VO_2_ peak: 16.01 ± 4.11 vs. 15.66 ± 4.14 vs. 13.33 ± 3.4 mL/min/kg; CO: 6.96 ± 2.34 vs. 6.87 ± 2.37 vs. 6.05 ± 1.79 L/min; HR: 126.44 ± 20.53 vs. 115.15 ± 18.78 vs. 109.07 ± 16.23 bpm, *P* < 0.05). NOCAD had significantly lower VO_2_ at anaerobic threshold (-1.35, 95%CI -2.16 - -0.54) and VO_2_ peak (-2.05, 95%CI -3.18 - -0.93) compared with healthy volunteers after adjustment. All IHD patients were associated with low stroke volume and inefficient gas exchange (*P* < 0.05).

**Conclusion:**

IHD with increasing atherosclerotic burdens were associated with impaired cardiac output and chronotropic response on CPET. NOCAD should be given more early prevention and rigorous follow-up.

## Introduction

Ischemic heart disease (IHD) manifests in numerous ways, from non-obstructive coronary disease (NOCAD) to obstructive coronary disease (OCAD). Coronary atherosclerotic burden measured using invasive and noninvasive anatomic imaging modalities has been consistently demonstrated to be a powerful independent prognostic determinant of risk for heart failure (HF) and death [1–3]. The presence of cardiac dysfunction may be indicative of severe coronary stenosis, though no sufficient data demonstrated dose-response relationship [1]. Strategies based on the anatomical structure (e.g., coronary angiography (CAG), coronary computed tomographic angiography(CTA)) are useful for patients with OCAD, but not for patients with NOCAD that lacking of evident stenosis for large coronary vessels [4]. The methods of functional evaluation, instead, can assess IHD from the perspective of cardiac function regardless of the stenosis severity [4, 5].

Cardiopulmonary exercise test (CPET) is a noninvasive and safe approach to assess cardiopulmonary function and helps understand underlying pathophysiological mechanisms [6]. CPET for IHD assessment is an area of growing clinical interests [7] since it provides a thorough assessment of exercise integrative physiology involving the pulmonary, cardiovascular, muscular, and cellular oxidative systems [8] Combination of cardiac electrophysiology variables (e.g., heart rate (HR) and electrocardiogram (ECG)) and gas exchange variables (e.g., oxygen uptake (VO_2_), O_2_ pulse (VO_2_/HR), VO_2_ relative to work-rate (VO_2_/WR)) [8–14] give clinicians unique insights on the evaluation of IHD [15–19]. The diagnostic and prognostic role of CPET in IHD have been confirmed in previous studies [7, 11, 13, 18, 20]. However, the difference of cardiac function on CPET in IHD with different coronary atherosclerotic burdens is unknown. The purpose of the study is to analyze cardiac functional characteristics of IHD on CPET with different coronary atherosclerotic burdens .

## Methods

### Study population

This was a cross-sectional observational study that included ischemic symptomatic patients in Beijing Tingshua Changgung Hospital from March 2018 to September 2019. The ischemic symptomatic patients that had either typical or atypical angina on Rose questionnaire [21] were divided into three groups according to the degree of coronary stenosis by CAG: (1) NOCAD: those that had ischemic symptoms but all the coronary arteries stenosis less 50%; (2) OCAD: those that had ischemic symptoms and had at least one of the coronary arteries stenosis from 50 to 90%; (3) severe OCAD: those that had ischemic symptoms and had at least one of the coronary arteries stenosis from 90 to 100%. Patients with a history of asthma, chronic obstructive pulmonary disease, hypertrophic cardiomyopathy, dilated cardiomyopathy, and valvular heart disease were excluded. In addition, we enrolled 101 healthy volunteers as controls that meeting the following criteria: > 18 years old; no symptoms of chest discomfort; no reported history of cardiovascular disease or pulmonary disease; no contradictions for CPET [6]. All participants provided written informed consent. The study was approved by the Research Ethics Committee of Beijing Tingshua Changgung Hospital.

### Cardiopulmonary exercise test

CPET was performed on a cycle ergometer (Miraclink-200P, China) within 1 month after CAG. The 12-lead ECG, HR, and blood pressure were continuously monitored using an automated sphygmomanometer (Tango M2, SunTech, USA) every 2 min during the test. Inhaled and expired gasses were collected by a face mask and analyzed breath-by-breath using the Geratherm Respiratory (Ergostik, Blue Cherry Software, Germany). All participants except for severe OCAD underwent the symptom-limited exercise test with the workload tailored to the individual’s age, height, weight, and exercise habit. Severe OCAD patients conducted the low-level exercise test with the incremental WR of 10 W/min. All subjects were asked to cycle at a constant rate of 60 rpm and encouraged to exercise until achieving a respiratory exchange ratio (RER) ⩾ 1.10 or HR peak ⩾ 85% predicted HR peak [22]. Peak values were expressed as the 30-second-average at the highest workload achieved [6]. Anaerobic threshold (AT) was defined as the moment where lactic acid production exceeded its removal, determined by the V slope method or, if unclear, by the ventilator equivalent method [6].

VO_2_, predicted% VO_2_ peak, VO_2_/WR slope, VO_2_/HR, estimated cardiac output (CO), RER, etc. were automatically calculated by the Blue Cherry Software. CO was calculated by dividingVO_2_ by the arterial-venous oxygen content difference ([C(a-v)O2]), using the Fick principle. Predicted HR peak was calculated as (220 – age) on no β-blocker therapy and (119 + 0.5× HR rest – 0.5× age) on β-blocker therapy. HR/WR was calculated as HR divided by WR at AT or peak, respectively. We constructed exercise/rest ratio (peak/rest, AT/rest) for the below variables: HR, VO_2_, VO_2_/HR, CO.

### Statistical analysis

Participants were compared between below groups: healthy volunteers, NOCAD, OCAD, severe OCAD. Continuous variables with normal distribution were reported as the mean ± SD and tested by one-way ANOVA while non-normal distribution reported as median and interquartile range (IQR) and tested by Wilcoxon rank-sum test. Categorical variables were reported as percentages. Among-group comparisons were made using a 𝞦^2^ test or a Fisher’s exact test if any expected cell count was less than five. Histograms were plotted to describe the distribution of CPET variables in healthy volunteers. 98% and 2% percentile were considered to be the lower and upper limits in our sample population.

We first modeled each CPET variable as the continuous variable separately against the four groups using the general linear model. Model 1 was the univariate model. Model 2 additionally adjusted for age and gender. Model 3 additionally adjusted for body mass index (BMI), compared with model 2. Model 4 additionally adjusted for use of HR limiting medication (e.g. metoprolol, diltiazem) compared with model 3. BMI was omitted from models for VO_2_ since they were already adjusted for weight. Age, gender, BMI were omitted from models for predicted % VO_2_ since they were already adjusted. HR and HR exercise/rest ratio were additionally adjusted for WR since we assumed it as the confounder. Adjusted LS-means with 95% CI were plotted for each group. Tukey-Kramer was used for adjustment for p-value for multi-groups comparison. For categorical CPET variables, the Chi-square test was used for univariate analysis. The logistic regression model for binary outcomes and polytomous logistic regression model for multi-nominal variables, adjusted for the aforementioned confounders, were constructed for multivariate analysis.

Participants were categorized by cardiac function and pathophysiological patterns. Cardiac function evaluation was defined according to EACPR/AHA statement [9]. Pathophysiological patterns were defined according to AHA evaluation paradigm [22]. Briefly, chronotropic insufficiency was defined as high VO_2_/HR and low peak HR; low stroke volume (SV) was defined as low VO_2_ peak or low VO_2_/WR slope or low VO_2_/HR; inefficient pulmonary gas change was defined as high VE/VCO_2_ or high Vd/Vt. Cut-off value for each variable was derived from reference value in our healthy participants.

We performed sensitivity analysis using the criteria below: (1) excluded participants with insufficient effort (i.e. RER < 1.10 or HR peak < 85% predicted HR peak); (2) excluded participants taking HR limiting medications; (3) adjusted for history of hypertension, diabetes and hyperlipidemia in addition to model 4.

Statistical analysis was carried out in SAS 9.4 (SAS Institute, Cary, NC, USA).

## Results

### Baseline characteristics

**Fig. 1:**
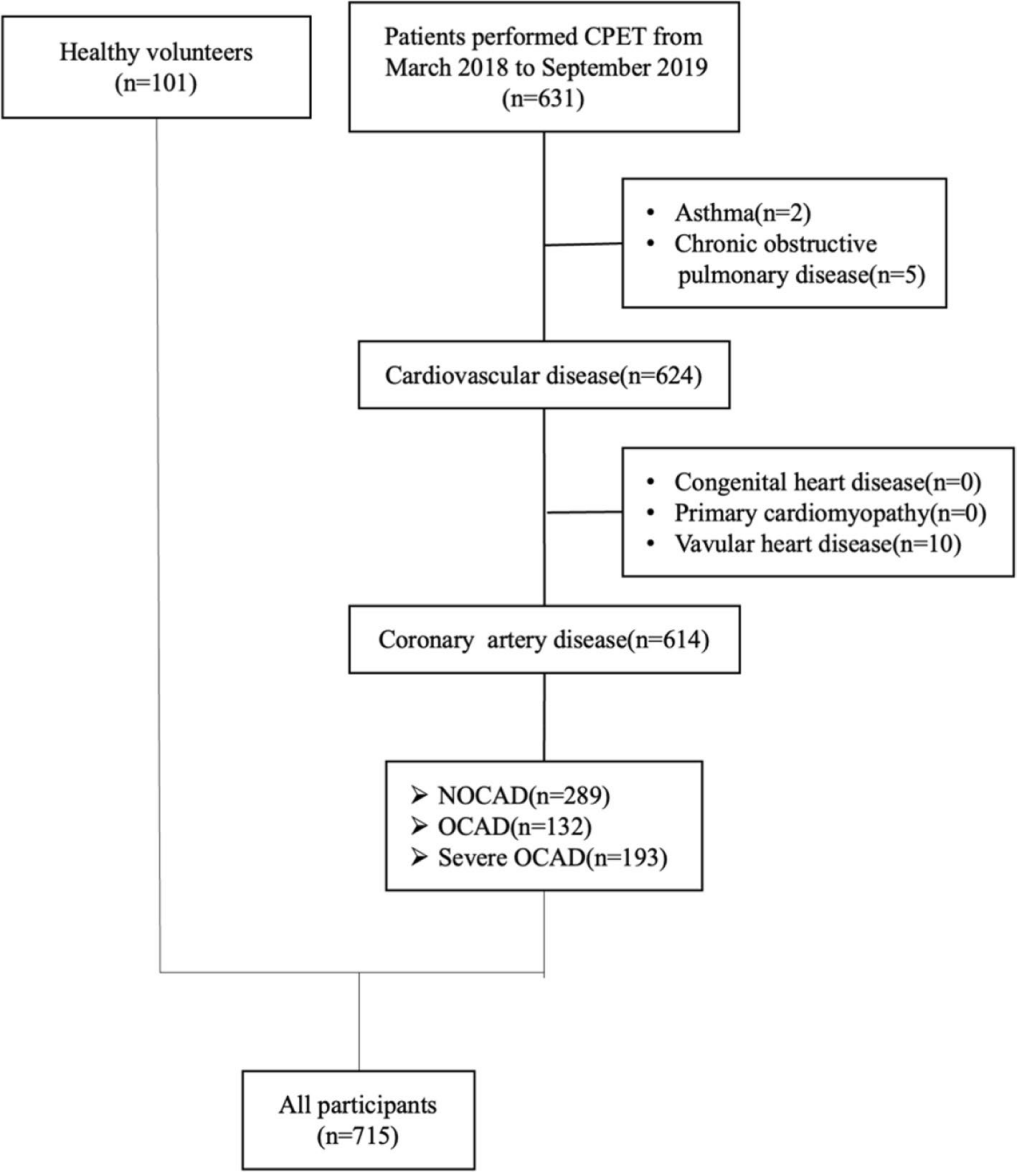
Flow chart of the study. CPET, cardiopulmonary exercise test; NOCAD, non-obstructive coronary artery disease; OCAD, obstructive coronary artery disease; AMI, acute myocardial infarction

The study population comprised of 614 patients with a mean age of 60 years and 101 healthy volunteers with a mean age of 37 years (Fig. 1). Severe OCAD patients were more younger, and had a higher BMI and HbA1c and high sensitivity cardiac troponin T and NT-proBNP, and had a higher incidence of hyperlipidemia compared with NOCAD and OCAD patients. NOCAD patients had the highest percent of women and the highest levels of low density lipoprotein cholesterol. And severe OCAD patients had the largest left ventricular end-diastolic dimension (LVEDD) and the lowest left ventricular ejection fraction (LVEF) (Table 1). There were 341 (47.7%) achieved RER ⩾1.1, 632 achieved HR peak ⩾ 85% predicted HR peak, a total of 669 (93.6%) participants achieved RER ⩾ 1.1 or HR peak ⩾ 85% predicted HR peak.

**Table 1:** Baseline characteristics of study population

Variables	Healthy	NOCAD	OCAD	Severe OCAD	***p*** **-value**
	(***N* = 101)**	(***N* = 289)**	(***N* = 132)**	(***N* = 193)**	
Age (year)	36.93 ± 12.15	60.11 ± 10.46	61.82 ± 10.45	57.14 ± 10.55	< 0.0001
Female, N (%)	59(58.42%)	161(55.71%)	34(26.36%)	42(22.11%)	< 0.0001
BMI (kg/m^2^)	23.4 ± 3.33	25.49 ± 3.36	25.16 ± 3.65	25.66 ± 3.35	< 0.0001
Hypertension, N (%)	0 (0%)	178 (62.02%)	96 (72.73%)	112 (58.64%)	< 0.0001
Hyperlipidemia, N (%)	0 (0%)	181 (63.07%)	105 (79.55%)	167 (87.43%)	< 0.0001
Diabetes, N (%)	0 (0%)	74 (25.78%)	54 (40.91%)	52 (27.23%)	< 0.0001
𝛃-blocker, N (%)	0 (0%)	56 (21.71%)	49 (40.83%)	116 (67.05%)	< 0.0001
Diltiazem, N (%)	0(0%)	9(3.48%)	6(5.00%)	5(2.19%)	< 0.0001
Haemoglobin (g/l)	134.77 ± 17.62	139.32 ± 13.8	136.7 ± 15.87	138.95 ± 15.77	0.21
hs-cTnT (ng/ml)	0(0,0.01)	0.01(0,0.01)	0.01(0.01,0.02)	0.05(0.01,0.36)	< 0.0001
NT-proBNP (pg/dl)	89(54,136)	57(25,97)	93(38,154)	269(110.5,587)	< 0.0001
Fasting glucose (mmol/l)	5.62 ± 0.9	5.9 ± 1.65	6.06 ± 1.59	6.31 ± 2.01	0.04
eGFR (ml/min*1.73m^2^)	107.46 ± 15.33	93.76 ± 14.31	88.29 ± 17.59	89.29 ± 16.51	< 0.0001
TC (mmol/l)	4.59 ± 0.86	4.6 ± 0.94	3.75 ± 0.87	4.13 ± 0.99	< 0.0001
TG (mmol/l)	1.52(0.96,2.14)	1.42(1.05,1.95)	1.24(0.88,1.8)	1.6(1.17,2.03)	0.25
HDL-C (mmol/l)	1.16 ± 0.24	1.15 ± 0.29	1.05 ± 0.26	0.93 ± 0.21	< 0.0001
LDL-C (mmol/l)	2.77 ± 0.87	2.81 ± 0.88	2.08 ± 0.66	2.58 ± 0.93	< 0.0001
HbA1c (%)	5.73 ± 0.56	6.12 ± 1.02	6.25 ± 0.86	6.45 ± 1.52	0.02
LVEDD (mm)	45.54 ± 3.37	46.65 ± 3.83	48.03 ± 4.07	49.33 ± 4.68	< 0.0001
LVEDV (ml)	95.79 ± 16.89	101.59 ± 19.72	108.76 ± 21.25	116.04 ± 26.12	< 0.0001
LVESD (mm)	26.75 ± 3.56	28.04 ± 3.42	29.63 ± 4.24	30.95 ± 5.11	< 0.0001
LVEF (%)	65.57 ± 2.86	65.43 ± 3.43	63.88 ± 5.39	58.46 ± 8.82	< 0.0001

*NOCAD* Non-obstructive coronary artery disease, *OCAD* Obstructive coronary artery disease, *severe OCAD* severe obstructive coronary artery disease, *BMI* Body mass index, *WBC* White blood cell count, *PLT* Platelet, *hs-cTnT* High sensitivity cardiac troponin T, *NT-proBNP* N-Terminal pro-brain natriuretic peptide, *eGFR* Estimated glomerular filtration rate, *TC* Total cholesterol, *TG* Total triglycerides, *HDL-C* High density lipoprotein cholesterol, *LDL-C* Low density lipoprotein cholesterol, *HbA1c* Glycosylated hemoglobin, *LVEDD* Left ventricular end-diastolic dimension, *LVEDV* Left ventricular end-diastolic volume, *LVESD* Left ventricular end-systolic dimension, *LVEF* Left ventricular ejection fraction

### Cardiac output in IHD

**Fig. 2:**
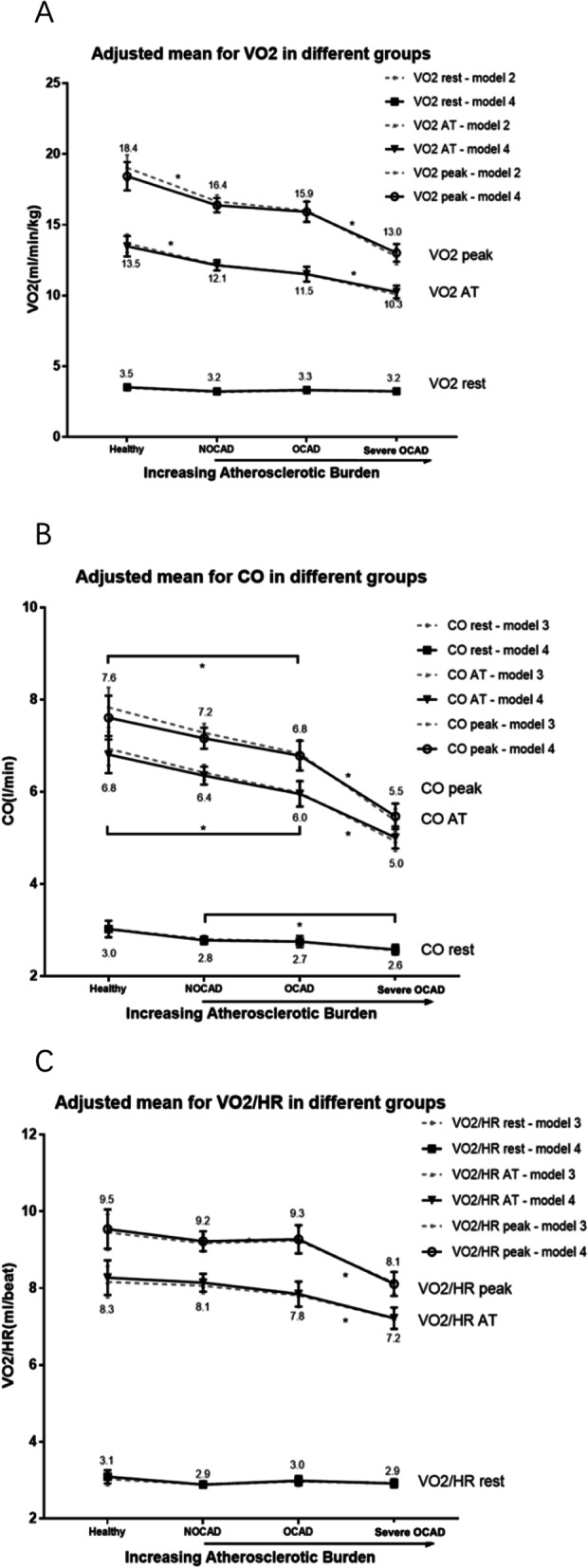
Variables associated with cardiac output. A.VO_2_ in different groups. VO_2_ AT, VO_2_ peak decreased with increasing atherosclerotic burden. VO_2_ AT and peak were significantly lower in NOCAD compared with healthy volunteers. B.CO in different groups. CO AT, CO peak decreased with increasing atherosclerotic burden, with a dramatic drop observed from OCAD. C. VO_2_/HR in different groups. VO_2_/HR decreased with increasing atherosclerotic burden and dramatically dropped in AMI patients. *: *P* < 0.05 in full-adjusted model. Only significant pairwise comparisons between the closest adjusted mean were shown in the figure

Generally, VO_2_ and CO in IHD were significantly lower than in healthy volunteers (Table 2). With increasing atherosclerotic burdens, significantly decreasing values of VO_2_ and CO at AT and peak were observed. Noticeably, compared with healthy volunteers, NOCAD patients had the worse CPET performance on variables like VO_2_, CO, VO_2_/HR. Multivariate analysis showed that with increasing atherosclerotic burdens, the decreasing trends for VO_2_ AT and VO_2_ peak were observed (*P* < 0.05). For pairwise comparison, we especially noticed a significant decrease of VO_2_ AT (β for NOCAD where the healthy as the reference, -1.35; 95% CI -2.16 - -0.54), VO_2_ peak (β for NOCAD where the healthy as the reference, -2.05; 95% CI -3.18 - -0.93) in NOCAD (Fig. 2A). And CO dropped dramatically in severe OCAD but only slightly declined in NOCAD (Fig. 2B). VO_2_/HR declined in severe OCAD patients but no significant difference was observed between NOCAD and healthy volunteers (Fig. 2C).

**Table 2:** Univariate analysis for CPET variables

Variables	Healthy	NOCAD	OCAD	Severe OCAD	***p***-value
	(***N* = 101)**	(***N* = 289)**	(***N* = 132)**	(***N* = 193)**	
**Resting value**					
VO_2_ rest (ml/min/kg)	3.71 ± 0.78	3.1 ± 0.57	3.22 ± 0.57	3.21 ± 0.56	< 0.0001
CO rest (l/min)	3.2 ± 1.36	2.73 ± 0.73	2.79 ± 0.68	2.76 ± 0.58	< 0.0001
HR rest (beats/min)	79.77 ± 11.38	75.73 ± 10.79	72.84 ± 10.56	73.75 ± 10.15	< 0.0001
VO_2_/HR rest (ml/beat)	2.96 ± 0.89	2.85 ± 0.79	3.08 ± 0.77	3.13 ± 0.7	0.0006
**AT value**					
VO_2_ AT (ml/min/kg)	15.21 ± 4.42	11.79 ± 2.74	11.33 ± 2.55	10.41 ± 2.37	< 0.0001
CO AT (l/min)	7.72 ± 2.89	6.15 ± 1.93	6.05 ± 1.86	5.51 ± 1.47	< 0.0001
HR AT (beats/min)	123.24 ± 18.89	105.03 ± 14.18	97.53 ± 13.05	96.98 ± 11.86	< 0.0001
VO_2_/HR AT (ml/beat)	8.12 ± 2.33	7.9 ± 2.25	8.17 ± 2.17	7.95 ± 1.95	0.62
HR/WR AT (beats/min/W)	1.51 ± 0.42	1.65 ± 0.56	1.59 ± 0.47	1.82 ± 0.64	< 0.0001
WR AT (W)	89.67 ± 34.38	69.62 ± 22.04	66.55 ± 20.43	58.14 ± 17.42	< 0.0001
**Peak Value**					
VO_2_ peak (ml/min/kg)	21.61 ± 5.83	16.01 ± 4.11	15.66 ± 4.14	13.33 ± 3.4	< 0.0001
Predicted%VO_2_peak (%)	66.96 ± 13.15	68.08 ± 15.09	62.02 ± 15.84	49.7 ± 13.23	< 0.0001
CO peak (l/min)	8.85 ± 3.16	6.96 ± 2.34	6.87 ± 2.37	6.05 ± 1.79	< 0.0001
Predicted% CO peak (%)	54.66 ± 10.68	51.19 ± 10.98	48.35 ± 12.2	40.18 ± 10.07	< 0.0001
HR peak (beats/min)	149 ± 22.96	126.44 ± 20.53	115.15 ± 18.78	109.07 ± 16.23	< 0.0001
VO_2_/HR peak (ml/beat)	9.52 ± 2.72	8.95 ± 2.62	9.6 ± 2.7	9 ± 2.28	0.03
HR/WR peak (beats/min/W)	1.2 ± 0.34	1.37 ± 0.46	1.32 ± 0.49	1.51 ± 0.47	< 0.0001
WR peak (W)	135.81 ± 46.49	102.55 ± 36.34	98.38 ± 36.91	79.19 ± 28.2	< 0.0001
VE/VCO_2_ slope	25.56 ± 3.95	28.89 ± 5.1	30.14 ± 5.65	29.68 ± 5.61	< 0.0001

*NOCAD* Non-obstructive coronary artery disease, *OCAD* Obstructive coronary artery disease, *severe OCAD* severe obstructive coronary artery disease, *VO<sub>2</sub>* Oxygen input, *CO* Cardiac output, *H*, Heart rate, *AT* Anaerobic threshold, *RER* Respiratory exchange rate, *WR* Work rate

#### Chronotropic response in IHD

**Fig. 3:**
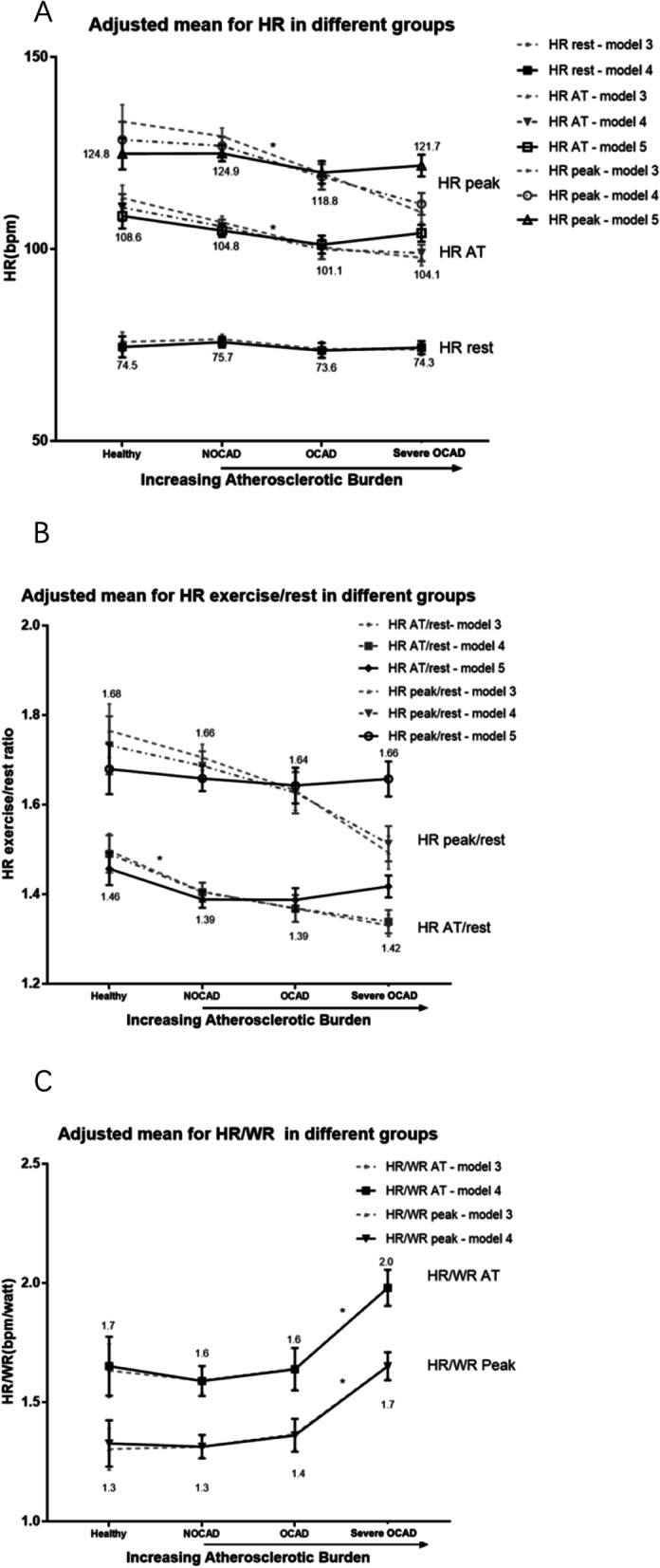
Variables with chronotropic response. **A** and **B** HR, HR exercise/rest ratio in different groups. No specific patterns observed with the increasing atherosclerotic burden after additionally adjustment of work rate. A significant decrease of HR AT/rest was noticed for NOCAD. C. HR/WR in different groups. An overall increasing pattern was noticed for HR/WR AT and peak, with increasing atherosclerotic burden. Both variable rocketed in AMI patients. *: *P* < 0.05 for full-adjusted model. Only significant pairwise comparisons between the closest adjusted mean were shown in the figure

HR in IHD was significantly lower than in healthy volunteers (Table 2). With increasing atherosclerotic burdens, a significantly decreased value of HR was observed (Table 2). Multivariate analysis showed that HR was lower for OCAD compared with NOCAD (Fig. 3 A); a significant decrease of HR AT/rest was observed for NOCAD (*P* < 0.05) (Fig. 3B); an overall increasing pattern was noticed for HR/WR (AT and peak), which especially rocketed in severe OCAD patients (Fig. 3 C).

### Pathophysiological patterns in IHD

**Fig. 4:**
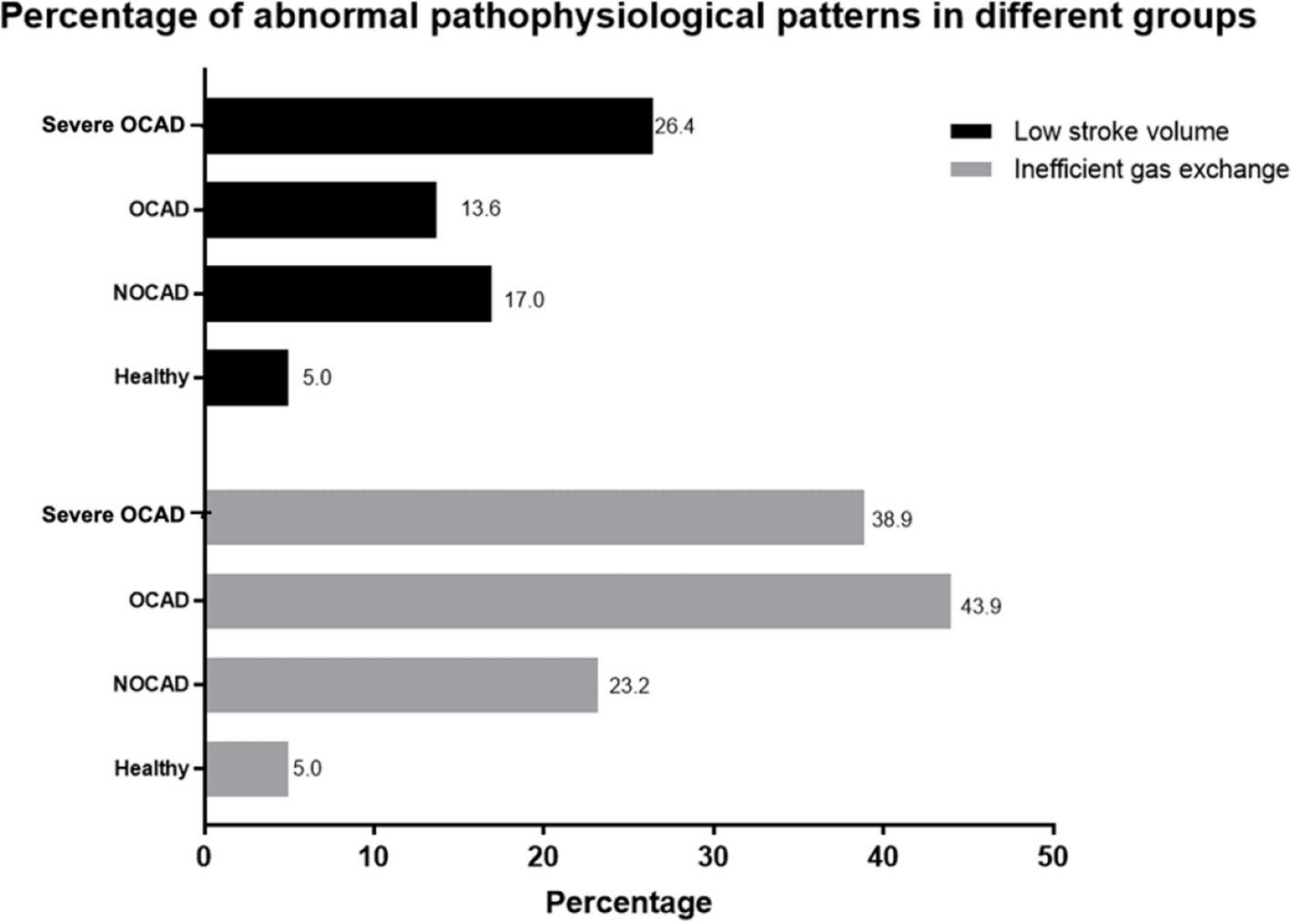
Distribution of abnormal pathophysiological patterns in different groups. Note : With healthy participants as controls, the abnormal pathophysiological patterns were compared in different groups. Low SV was defined as low VO_2_ peak or low VO_2_/WR slope or low VO_2_/HR; inefficient pulmonary gas change was defined as high VE/VCO_2_ or high Vd/Vt. In healthy participants, the mean value of VO_2_ peak was 21.61ml/min/kg, and the mean value of VO_2_/WR slope was 8.77, and the mean value of VO_2_/HR peak was 9.52, and the mean value of VE/VCO_2_slope was 25.56, and the mean value of Vd/Vt peak was 0.19

Generally, compared with healthy volunteers, participants with IHD had much higher frequency of abnormal circulatory impairment pattern (Fig. 4). All IHD patients were overall associated with low SV and inefficient gas exchange (*P* < 0.05). For distinctive groups, only severe OCAD was associated with low SV associated with inefficient gas exchange (Table 3). No significant results were observed for OCAD or NOCAD in adjusted model.

**Table 3:** Odds ratio for potential pathophysiological patterns

		NOCAD		OCAD		Severe OCAD	
	Model	OR (95%CI)	p*	OR (95%CI)	p*	OR (95%CI)	p*
Low stroke volume	1	3.92(1.52,10.14)	0.0048	2.91(1.04,8.19)	0.0426	6.86(2.64,17.82)	< 0.0001
	2	1.76(0.61,5.04)	0.2952	1.53(0.48,4.91)	0.477	4.73(1.63,13.68)	0.0042
	3	1.81(0.62,5.26)	0.2751	1.57(0.48,5.08)	0.4537	4.87(1.66,14.26)	0.0039
	4	2.77(0.75,10.24)	0.1273	2.61(0.64,10.61)	0.1802	6.81(1.78,26.08)	0.0051
Inefficient gas exchange	1	5.79(2.26,14.82)	0.0002	15.2(5.8,39.83)	< 0.0001	12.52(4.87,32.2)	< 0.0001
	2	1.04(0.37,2.95)	0.9384	1.93(0.65,5.69)	0.2336	2.21(0.78,6.22)	0.1337
	3	1.33(0.47,3.82)	0.5928	2.4(0.8,7.21)	0.1186	2.82(0.99,8.04)	0.0525
	4	1.33(0.41,4.28)	0.6367	2.21(0.65,7.49)	0.2024	2.96(0.91,9.67)	0.0728

*Healthy participants as reference group. Model 1 was univariate model. Model 2 additionally adjusted for age and gender. Model 3 additionally adjusted for BMI, compared with model 2. Model 4 additionally adjusted for use of HR limiting medications

## Discussion

To our knowledge, this is the first study that comprehensively compared the characteristics of cardiac function on CPET among NOCAD, OCAD, and severe OCAD patients. In this study we found: (1) with increasing atherosclerotic burdens, patients had the impaired cardiac output (VO_2_, CO) and chronotropic response (HR, HR exercise/rest ratio); (2) significant differences between NOCAD and healthy volunteers were noticed for VO_2_ and HR AT/rest in pairwise comparison after adjustment for confounders; (3) IHD patients were overall associated with low SV and inefficient gas exchange, especially severe OCAD. CPET is a useful tool to evaluate the cardiac function in different atherosclerotic burdened IHD that should be implied in the clinic to guide the management and treatment of IHD. Especially, for NOCAD patients, even if there is no further microcirculation anatomical evidence, cardiac functional evaluation by CPET can be used to stratify the cardiac risk. For NOCAD patients with abnormal functions, early prevention and rigorous follow-up are important measures to decrease the adverse cardiac events.

Cardiac function is manifested in cardiac output and chronotropic response. Some key variables were used as surrogates for cardiac output per minute, stroke volume as well as a direct measure of HR response [7]. In patients with cardiac dysfunction, myocardial oxygen deficit during exertion- induced mechanical dysfunction exceed the ischemic threshold, resulting in stroke volume to decrease with the progressively increasing workload. Therefore, the sympathetic activity up-regulated to accelerate HR as a compensation mechanism. The abrupt plateau or decrease in stroke volume was accompanied by a decrease in cardiac output, reflected by VO_2 _[9–13]. A more blunted VO_2_ response was consistent with cardiac severe status [7]. In this study, we compared the difference of the cardiac function by CPET in IHD patients with different atherosclerotic burdens (NOCAD, OCAD, severe OCAD). In our IHD patients, the performance on cardiac output (VO_2_, CO, VO_2_/HR) and chronotropic response (HR) on CPET worsened with increasing atherosclerotic burdens (severe OCAD worst, OCAD moderate, NOCAD best). Because of atherosclerotic burden, the exercise induced ischemia leaded to the acute decrease of cardiac output on CPET in our IHD patients.

Few studies focused on cardiac function assessed by CPET in different coronary atherosclerotic burdened IHD. Akinci Ozyurek et al [23] selected subjects with chest pain to undergo CPET and CAG, and found that peak VO_2_ and VO_2_/HR were higher in patients with normal angiographic results than those with OCAD, though without statistical significance; HR peak was higher in subjects with OCAD than in subjects without OCAD. Hassan Khan et al [24] reported that peak VO_2_ was significantly lower in OCAD than in participants without OCAD (28.3 ± 8.1 VS 31.2 ± 7.7 ml/kg/min, *P* < 0.01) in the Kuopio Ischemic Heart Disease Risk Factor Study. Ellen Coeckelberghs et al [18] reported that 1409 IHD patients composed of AMI and OCAD had peak VO_2_ 19.5 ± 5.6 ml/kg/min and 73 ± 17% of predicted and peak HR 124 ± 21 bpm. Bong-Joon Kim et al [25] concentrated on the elderly patients with cardiovascular disease in Korea that showed an average exercise capacity of 21.3 ± 5.5 ml/kg/min at peak VO_2_, and men showed better exercise capacity than women on most CEPT parameters. Compared with previous studies, [18, 23–25] our IHD patients in different coronary atherosclerotic burdens generally had a lower levels of VO_2_ peak (16.01 ± 4.92 ml/kg/min), predicted% VO_2_ peak (61.84 ± 16.38%), VO_2_/HR (9.17 ± 2.57 ml/beat) that may be partially attributed to racial differences.

VO_2_ peak, recognized as cardiorespiratory fitness, [15] was proposed as a vital indicator of prognosis in IHD [26]. Declined VO_2_ peak was related to lower levels of aerobic capacity and could indicate subclinical pathophysiology [16]. Increased VO_2_ peak could have substantial benefits in reducing the burden of IHD [27]. Chaudhry [28] illustrated a considerable decline in VO_2_ peak and VO_2_/HR in male NOCAD and a slight decrease in female NOCAD. VO_2_ peak and VO_2_ AT were significantly attenuated in cardiac syndrome X, [29] and women with NOCAD had markedly reduced VO_2_ peak [20]. In our study, VO_2_ at AT and peak decreased with increasing atherosclerotic burdens that suggested a deterioration of cardiac function in our IHD patients. In accord with previous studies, our NOCAD patients had a lower VO_2_ than healthy participants that may related to the microcirculation dysfunction should be emphasized to trace the cardiovascular risk factors to early prevent and follow up.

Chaudhry [28] observed a pathological HR response in NOCAD and abnormal HR response was more effective than stress ECG test for identifying cardiac dysfunction. HR/WR slope reclassified abnormalities in the NOCAD from 22 to 81%. In our study, a significant difference of HR/WR between NOCAD and healthy participants was not detected after adjustment. HR/WR was defined as the value at a specific time (AT, peak) in our study, which was not exactly the change in HR as a function of WR in the last 2 min of exercise as Chaudhry’s study. Particularly, the greater decline of WR relative to HR in NOCAD might lead to a slightly increased ratio of HR/WR, which implied an impaired exercise capacity. More study should focus on the impact of WR on performance of CPET and the complex role of other compounders factors in CPET.

NOCAD was a marker of the more adverse risk factor profile [30]. CPET was also used to expand the role in microvascular coronary heart disease, beyond identifying flow-limiting lesions [31]. Subjects with either macrovascular or microvascular coronary heart disease could demonstrate a similar CPET response, although cardiac catheterization findings may be different [31]. Thus, we applied CPET to evaluate the NOCAD patients in our study. It was noticeable that compared with healthy volunteers, some NOCAD patients performed worse even after adjustment, namely, lower VO_2_ peak, and HR response. More studies demonstrated that abnormal dilatory responses of the coronary microvessels and coronary microvascular spasm were identified as pathogenic mechanisms in both chronic and acute forms of ischemic heart disease [5, 32]. It was supposed that the microcirculation dysfunction was related to the decrease of coronary oxygen uptake and cardiac output that manifesting as lower VO_2_ and CO in NOCAD compared to the healthy participants. We extrapolated those NOCAD patients to have impaired cardiac function and poor prognosis. Though invasive microvascular function tests were not conducted, CPET variables still conveyed substantial information for evaluation of the function and prognosis in NOCAD. And the demonstration of coronary microvascular dysfunction in NOCAD (i.e., reduced coronary flow reserve or microvascular spasm) can be investigated during angiography using intracoronary adenosine and ACh.

Our study has several limitations: (1) This is a single-center study that may introduce selection bias. (2) Healthy participants were younger than symptomatic patients. We were not able to enroll enough age and gender-matched symptomatic and healthy participants. But we adjusted for age in the study and help to reduce the impact of confounding.

## Conclusion

Associations were observed between increasing atherosclerotic burdens and unfavorable CPET variables for cardiac output and chronotropic response. NOCAD patients had a lower VO_2_ peak and HR response compared with healthy volunteers that should be given more early prevention and rigorous follow-up. CPET can be a useful tool to evaluate cardiac function in different atherosclerotic burdened heart diseases.

## Data Availability

The datasets generated and analyzed during the current study are not publicly available due privacy and ethical restrictions but are available from the corresponding author on reasonable request.

## Abbreviations

BMI: Body mass index
CAG: Coronary angiography
[C(a-v)O2]: Arterial-venous oxygen content difference
CO: Estimated cardiac output
CPET: Cardiopulmonary exercise test
CTA: Coronary computed tomographic angiography
ECG: Electrocardiogram
HR: Heart rate
IHD: Ischemic heart disease
LVEDD: Left ventricular end-diastolic dimension
LVEF: Left ventricular ejection fraction
NOCAD: Non-obstructive coronary artery disease
OCAD: Obstructive coronary artery disease
SV: Stroke volume
VO_2_: Oxygen uptake
